# Decoupling the role of stiffness from other hydroxyapatite signalling cues in periosteal derived stem cell differentiation

**DOI:** 10.1038/srep10778

**Published:** 2015-06-02

**Authors:** Giorgio Mattei, Concetta Ferretti, Annalisa Tirella, Arti Ahluwalia, Monica Mattioli-Belmonte

**Affiliations:** 1 Research Centre “E. Piaggio”, University of Pisa, Largo Lucio Lazzarino 1, 56122 Pisa, Italy; 2 Department of Clinical and Molecular Sciences-Histology, Università Politecnica delle Marche, Via Tronto 10/A, 60020 Ancona, Italy; 3 National Research Council, IFC, Via Moruzzi 1, 56124 Pisa, Italy

## Abstract

Bone extracellular matrix (ECM) is a natural composite made of collagen and mineral hydroxyapatite (HA). Dynamic cell-ECM interactions play a critical role in regulating cell differentiation and function. Understanding the principal ECM cues promoting osteogenic differentiation would be pivotal for both bone tissue engineering and regenerative medicine. Altering the mineral content generally modifies the stiffness as well as other physicochemical cues provided by composite materials, complicating the “cause-effect” analysis of resultant cell behaviour. To isolate the contribution of mechanical cues from other HA-derived signals, we developed and characterised composite HA/gelatin scaffolds with different mineral contents along with a set of stiffness-matched HA-free gelatin scaffolds. Samples were seeded with human periosteal derived progenitor cells (PDPCs) and cultured over 7 days, analysing their resultant morphology and gene expression. Our results show that both stiffness and HA contribute to directing PDPC osteogenic differentiation, highlighting the role of stiffness in triggering the expression of osteogenic genes and of HA in accelerating the process, particularly at high concentrations.

Bone is one of the most transplanted tissues, with more than 500,000 graft procedures performed yearly in the United States and 2.2 million worldwide^1^. Although autografts currently represent the “gold standard”, they are limited in size, shape and availability^2^,^3^. Moreover, they often result in donor site morbidity, especially in the elderly^2^,^4^,^5^. Allografts are generally less affected by size and shape constraints, but are limited by donor tissue availability and increased risk of immunogenic response, disease transmission and infection^3^. These limitations have prompted extensive research and commercial development for synthetic bone graft substitutes. The scaffold plays a critical role in the successful engineering of bone tissue, providing a suitable environment for cell migration, proliferation and differentiation towards the desired tissue phenotype. The consensus is that the ideal scaffold for bone regeneration – as well as for other tissues – should mimic the morphology, structure, physicochemical properties and function of the native tissue^6^. A bone scaffold should also be osteoconductive (to recruit bone cells from the recipient), osteoinductive (to differentiate stem cells into bone-forming cells) and capable of promoting osteointegration (to provide permanent and functional attachment to native bone)^7^. Bone is a natural composite material containing about 70% mineral phase (mainly hydroxyapatite, HA) and 30% organic phase^8^. The latter is composed of 98% bone matrix (mainly collagen) and 2% bone cells (i.e. osteoblasts, osteocytes and osteoclasts). Therefore, HA crystals and natural polymer (e.g. collagen, gelatin, chitosan) composites have been widely investigated to develop biomimetic scaffolds for bone tissue engineering applications^9^,^10^,^11^,^12^,^13^,^14^,^15^,^16^,^17^. Gelatin (a commercially available biomaterial derived from collagen) is often used as the organic component of scaffolds because of its biocompatibility and capacity to promote cell adhesion and migration thanks to its Arg-Gly-Asp (RGD)-like sequences. Gelatin-based scaffolds are often covalently crosslinked using various chemical reagents (e.g. glutaraldehyde^18^,^19^, genipin^13^,^16^,^17^) or enzymes (e.g. transglutaminase^20^) to improve their stability and enhance the mechanical properties. The structural stability of the organic component can be further increased by the addition of a mineral-based filler. Several reports in the literature show that the use of HA particles (or any other inorganic component), with a constant amount of organic matrix phase increases the mechanical strength of composite bone scaffolds^13^,^16^,^17^,^21^,^22^,^23^. It is worth noting that the introduction of HA not only increases scaffold stiffness, but also alters other physicochemical cues provided by the inorganic phase.

Changes in mineralisation, collagen crosslinking and bone density all appear to contribute to modifications in the bone microenvironment with time, although it is still unclear how age affects the integrative material properties of osteal tissue^24^. Ageing bone is characterised by a decrease in mechanical strength accompanied by alterations in stiffness and mineral content, with a concomitant decrease in osteoregenerative potential^25^. To engineer novel biomaterials with an optimal stiffness and with mineral content and organic component capable of directing osteoregeneration, while limiting material-dependent senescent signals which may accelerate age-dependent processes, it would be useful to investigate the individual role of different environmental cues in modulating cell behaviour. In this study, human periosteal derived progenitor cells (PDPCs) were used as a model of bone precursor cells. The periosteum plays a key role in bone repair^26^ and PDPCs, which reside in its inner cambium layer^27^, are able to differentiate into osteochondral cell types *in vitro*^28^. Due to its external localisation on bone, periosteum is particularly sensitive to mechanical stimuli and, even in absence of other factors, mechanical load induces new bone formation from periosteum^29^, suggesting that this is a highly specialised mechano-sensing tissue^30^. The ability of PDPC to carry intracellular tension through their microfilament network modulates bone and cartilage growth^30^, making them suitable for bone regeneration strategies^31^ as well as for the development of *in vitro* bone tissue models.

To isolate the specific contribution of mechanical cues from the effects of other bulk and surface HA-related signals (e.g. roughness, mineral content) in modulating progenitor cell differentiation towards an osteogenic phenotype we designed composite hydroxyapatite/gelatin (HA/Gel) hydrogel scaffolds containing different amounts of inorganic phase and a parallel set of stiffness-matched HA-free gelatin scaffolds (Gel) crosslinked with various concentrations of glutaraldehyde (GTA)^32^,^33^,^34^. The stiffness of Gel scaffolds was correlated with GTA concentration, while that of HA/Gel scaffolds was related to the HA content. The scaffolds were then seeded with human PDPCs and cultured over 7 days to investigate their differentiating potential. Cell morphology was analysed by scanning electron microscopy (SEM), while mRNA levels of genes expressed during the several phases of osteoblast differentiation were selectively assessed using quantitative real time PCR (qRT-PCR). We show here that PDPC differentiation is modulated both by HA and substrate stiffness, but HA’s physicochemical signalling is a far more important trigger for osteogenic differentiation at high concentrations than substrate stiffness.

## Results

### Scaffold mechanical properties

**Figure 1:**
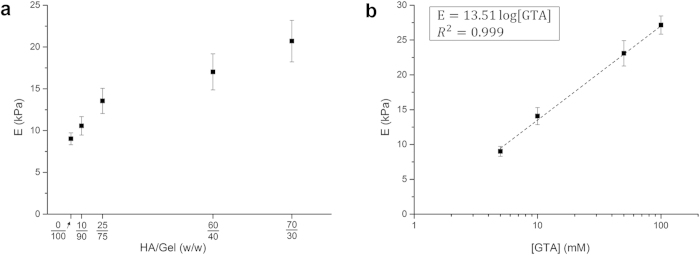
Compressive moduli obtained for (**a**) HA/Gel and (**b**) Gel scaffolds. Results are reported as a function of HA/Gel weight ratio and GTA concentration, respectively.

Compressive moduli of HA/Gel and Gel hydrogels, expressed respectively as a function of HA/Gel weight ratio and GTA concentration, are shown in Fig. 1. As expected the bulk compressive modulus of HA/Gel scaffolds increases significantly with HA content (Fig. 1a). Similarly, since the number of crosslinks formed depends on crosslinker concentration, Gel scaffold stiffness increases with GTA (Fig. 1b). In particular, the compressive modulus (*E*) of Gel scaffolds follows a logarithmic-trend with GTA concentration: in a semi-log domain a linear relation was determined as *E* = 13.51∙log[GTA] (linear regression, R^2^ = 0.999).

On the basis of this relationship, Gel scaffolds were consequently crosslinked with 6, 10, 18 and 34 mM GTA solutions, as mechanical equivalents of the 10/90, 25/75, 60/40 and 70/30 HA/Gel samples. To facilitate the comparison between HA/Gel samples and their mechanical equivalents (Gel), the latter were coded using the same HA/Gel ratio of the respective HA-containing samples followed by “Gel” (i.e. 10/90, 25/75, 60/40 and 70/30 Gel). The 0/100 HA/Gel is the same as 0/100 Gel, and was considered as the control.

### Cell characterisation

**Figure 2:**
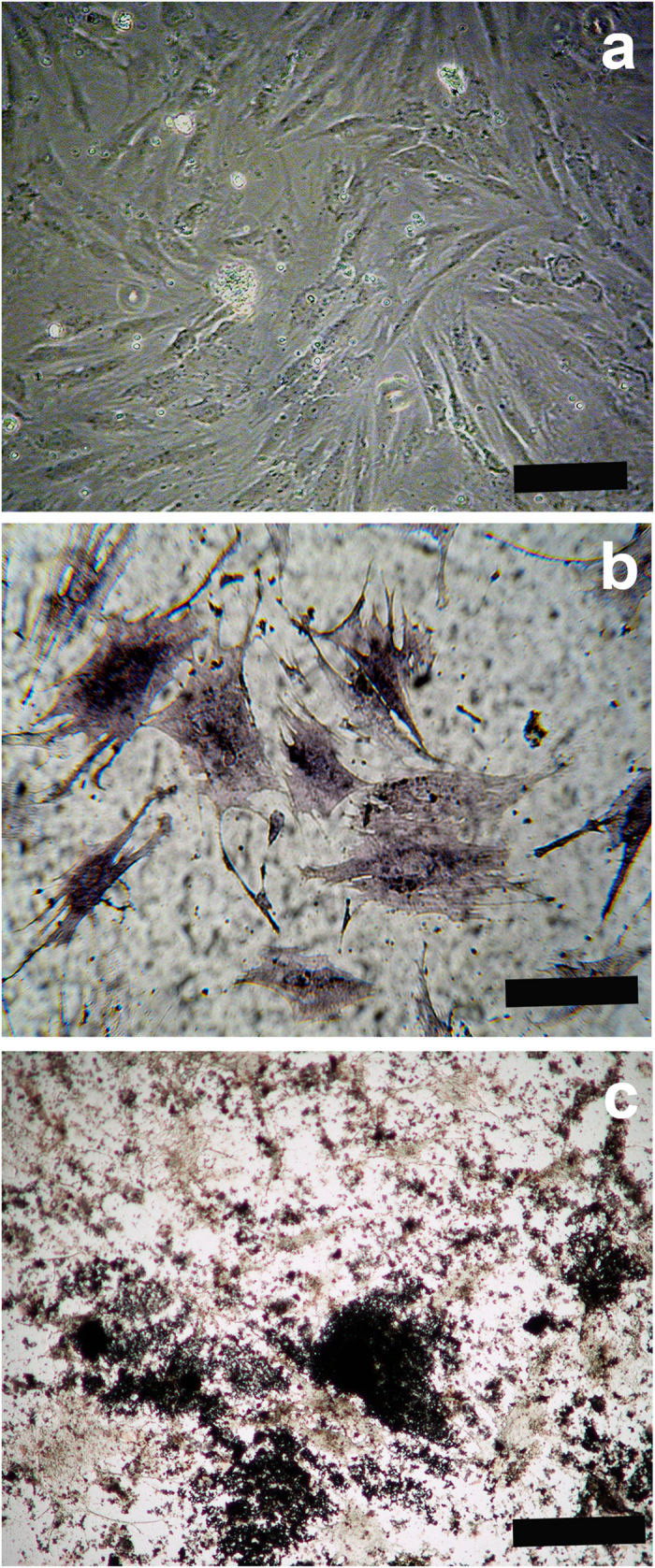
(**a**) Fibroblast-like morphology of PDPCs observed in phase contrast, (**b**) Osteogenic differentiation of PDPCs assessed by ALP staining, and (**c**) identification of CaPO_4_ crystals by Von Kossa reaction. Scale bars: (**a**) 10 μm; (**b**) and (**c**) 20 μm.

**Figure 3:**
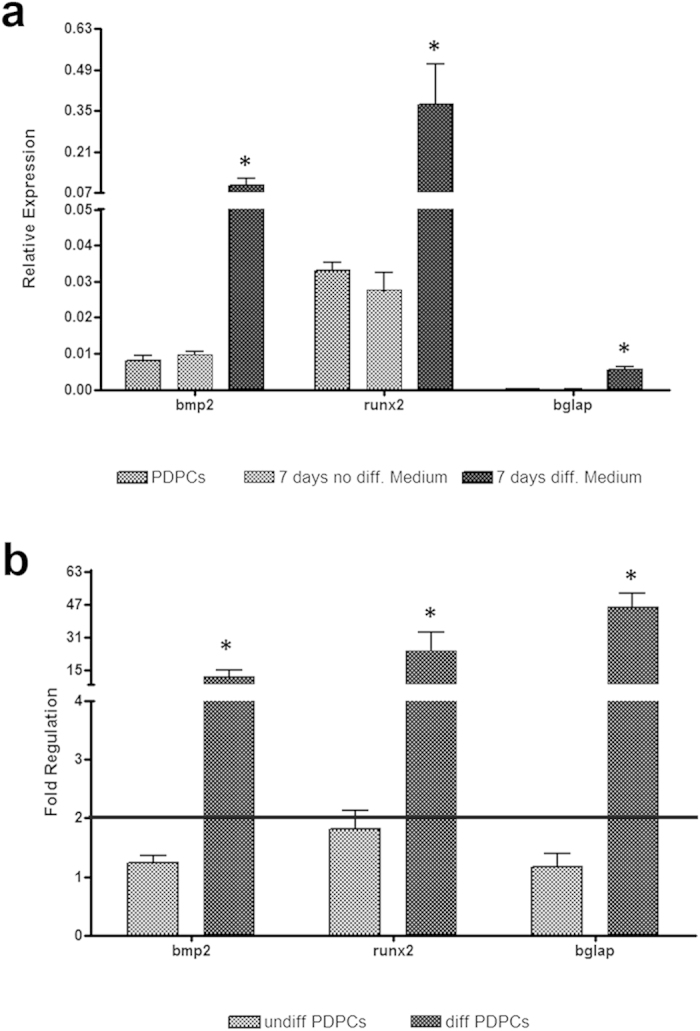
Histograms depict changes between PDPC basal gene expression (control) of bmp2, runx2 and osteocalcin (bglap) and that observed after culturing cells for 7 days on TCP either using DMEM/F12 non-differentiating medium or osteogenic differentiating medium. Data are expressed either as relative expression (**a**) or as fold-regulation with respect to PDPCs control (**b**), which represents fold-change results in a biologically expressive manner. In particular, the fold-regulation is equal to the fold-change (2^−ΔΔCt^) for fold-change values greater than one, which indicate an up-regulation. Fold-change values less than one indicate a down-regulation: in this case the fold-regulation is the negative inverse of the fold-change (−1/2^−ΔΔCt^). The black line in (**b**) indicates the range of physiological mRNA expression changes (i.e. fold-regulation < 2). Statistical differences between undifferentiated and differentiated PDPCs are denoted with an asterisk (* *p* < 0.05).

PDPCs resulted positive for CD73, CD90 and CD105 and negative for CD45, HLA-DR and CD14 in agreement with the criteria of the International Society for Cytotherapy (Supplementary Fig. 1). Phase-contrast microscopy highlighted the fibroblast-like morphology of PDPCs cultured on tissue culture plates (TCPs) (Fig. 2a). This feature changes after their treatment (7 days) in an osteogenic differentiating medium, with cells acquiring a prevalent star-shaped morphology. Moreover, after differentiation PDPCs became strongly positive for alkaline phosphatase (Fig. 2b) and aggregates of mineralized matrix were determined by Von Kossa staining (Fig. 2c). Relative gene expression data are summarised in Fig. 3, which reports PDPCs basal expression (control) and that observed culturing cells for 7 days on TCP either in non-differentiating medium (i.e. DMEM/F12) or osteogenic differentiating medium. No changes between basal gene expression and that after 7 days of culture in DMEM/F12 were observed for all the genes analysed. On the contrary, a significant increase in the expression of all genes involved in osteogenesis was observed after culturing PDPCs in osteogenic differentiating medium for 7 days with respect to their basal expression (Fig. 3a). The comparison between gene expression of PDPCs cultured in non-differentiating and osteogenic media are more evident using the ΔΔCt method, reporting results as fold-regulation with respect to basal gene expression (Fig. 3b).

### Scanning electron microscopy (SEM) and micro-analytical investigation (EDX)

**Figure 4:**
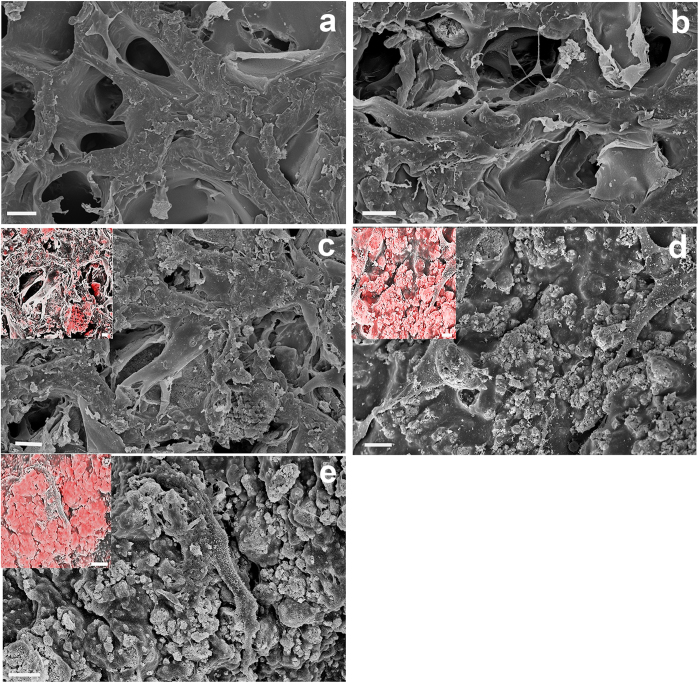
SEM micrographs of PDPCs cultured for 7 days on the different HA/Gel scaffolds. (**a**) 0/100; (**b**) 10/90; (**c**) 25/75; (**d**) 60/40; (**e**) 70/30. Inserts show SEM/EDX mapping of the different distribution of Ca^2+^ ions (red) in 25/75, 60/40 and 70/30 HA/Gel scaffolds. SEM/EDX mapping for 0/100 and 10/90 HA/Gel samples was not achieved due to the absence or the very low concentration of HA, respectively. Scale bars 10 μm.

**Figure 5:**
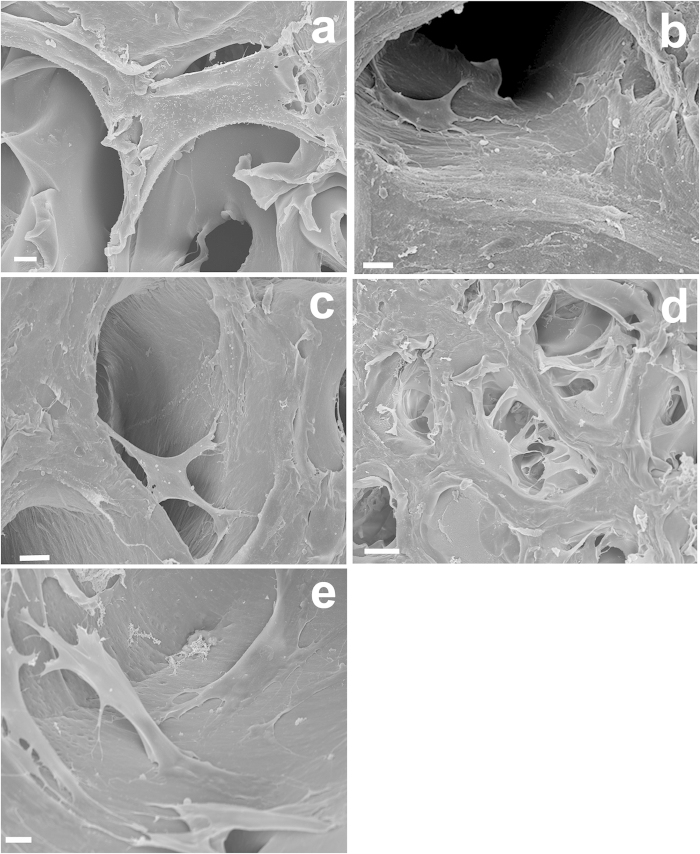
SEM micrographs of PDPCs seeded for 7 days on the different Gel scaffolds. (**a**) 0/100; (**b**) 10/90; (**c**) 25/75; (**d**) 60/40; (**e**) 70/30. Scale bars 10 μm.

After 2 days PDPCs were able to adhere on all the scaffolds, independent of their composition, with minimal differences in cell morphology (data not shown). At day 7, good scaffold colonisation was observed with changes in cell morphology related to the presence of HA. In particular, on HA/Gel scaffolds the increase in HA content from 25/75 to 70/30 affected cell spreading, with cells exhibiting a more elongated shape (Fig. 4). The SEM/EDX mapping confirmed an increase of calcium content in these scaffolds (as shown in Fig. 4 inserts). On the contrary, cells adopted a star-shaped morphology on all Gel scaffolds, with no significant differences related to stiffness variations (Fig. 5).

### RT-qPCR

**Figure 6:**
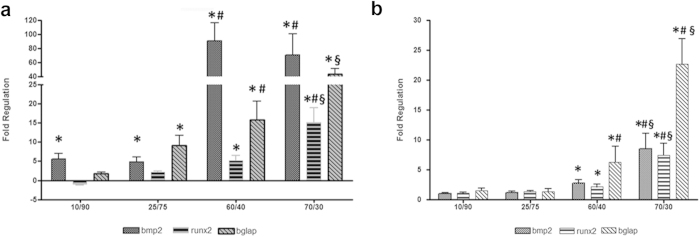
Changes in bmp2, runx2 and osteocalcin (bglap) mRNA expression in PDPCs seeded on (**a**) Gel and (**b**) HA/Gel scaffolds. Data are expressed as fold-regulation which represents fold-change results in a biologically expressive manner. Fold-regulation is equal to the fold-change (2^−ΔΔCt^) for fold-change values greater than one, which indicate an up-regulation. Fold-change values less than one indicate a down-regulation: in this case the fold-regulation is the negative inverse of the fold-change (−1/2^−ΔΔCt^). a = *p* < 0.05 vs CTRL; b = *p* < 0.05 vs 25/75 scaffolds; c = *p* < 0.05 vs 60/40 scaffolds.

RT-qPCR data are reported in Fig. 6 as fold-regulation over PDPCs seeded on 0/100 HA/Gel structures. In PDPCs cultured on HA/Gel scaffolds, significant (*p* = 0.0037) changes in bmp2 mRNA expression were detected for all tested scaffolds in comparison with the control, with the highest regulation in cells seeded on the 60/40 and the 70/30 HA/Gels (Fig. 6a). In these cells a significant increase in runx2 mRNA was also observed, with higher values in PDPCs seeded on the 70/30 HA/Gel than in cells cultured on 60/40 HA/Gel structures. As far as osteocalcin (bglap) mRNA expression is concerned, significantly higher levels were detected in cells seeded on 25/75, 60/40 and 70/30 HA/Gel scaffolds, with values greater than 15 fold with respect to the control in the latter two samples. Interestingly, in PDPCs seeded on 70/30 HA/Gel, the significant increase in bglap expression in comparison with 60/40 HA/Gel (22.63 ± 4.33 vs 6.23 ± 2.7, *p* = 0.039) was concomitant with a significant decrease in bmp2 (70.86 ± 29.87 vs 91.08 ± 25.35; *p* = 0.043), suggesting an acceleration in cell differentiation.

In PDPCs seeded on gelatin scaffolds, significant (*p* = 0.032) changes in all genes involved in osteogenesis (i.e bmp2, runx2 and bglap) were detected in cells seeded on 60/40 and 70/30 Gels in comparison with the 0/100 Gel, while no significant changes were observed for PDPCs on 10/90 and 25/75 Gels (Fig. 6b). Osteocalcin values higher than 15 fold were detected only in PDPCs seeded on 70/30 Gel.

Analysis of variance was also carried out between the HA/Gels and their mechanical Gel equivalents. In the presence of HA, all tested structures show significant differences in bmp2 mRNA expression. Moreover, significant differences in runx2 mRNA expression were detected in 60/40 (5.23 ± 1.31 vs 2.13 ± 0.43; *p* = 0.025) and 70/30 (15.15 ± 3.84 vs 7.43 ± 2.04; *p* = 0.017) HA/Gels. In addition, a significant up regulation of bglap was detected in cells seeded on 25/75 (9.13 ± 2.61 vs 1.32 ± 0.50; *p* = 0.017), 60/40 (15.78 ± 4.93 vs 6.23 ± 2.70; *p* = 0.025) and 70/30 (43.71 ± 7.50 vs 22.63 ± 4.33; *p* = 0.0015) HA/Gel scaffolds, in comparison with the corresponding HA-free Gel mechanical equivalents.

## Discussion

To decouple the contribution of mechanical cues from the effects owed to other HA signals in directing cell response, a range of stiffness-matched HA/gelatin (HA/Gel) and gelatin based (Gel) materials were developed and characterised. Glutaraldehyde was used as crosslinking agent at biocompatible concentrations, well below cytotoxic levels, to obtain stable materials and modulate Gel stiffness. Firstly, keeping GTA concentration constant, different quantities of HA were added to gelatin in order to mimic the physiological composition of osteochondral tissue. The quantity of HA filler over gelatin content ranged from 0/100 to 70/30 weight ratio covering cartilage (0/100 w/w) to subchondral bone (70/30 w/w)^11^,^13^,^35^. As expected, an increase in HA was accompanied by an increase in stiffness. The mechanical properties of gelatin-based materials were then characterised as a function of GTA. Based on the extensive literature on cell response to stiffness using substrates with different degrees of crosslinking^36^,^37^,^38^ as well as the experimental evidence on surface topography reported in the Supplementary Information SI 1, we assume that surface roughness is constant among Gel substrates. Moreover, since the Gel substrates contain the same amount of organic phase, the gelatin-related substrate properties can be considered constant among Gel samples. Having established a logarithmic relationship between scaffold stiffness and GTA concentration, crosslinking solutions of 6, 10, 18 and 34 mM GTA were used to obtain gelatin hydrogels matching the stiffness of HA/Gel samples. We underline that all samples contain the same amount of organic phase (i.e. 5% w/v of gelatin), hence HA/Gels and their matching Gel substrates differ in all HA-related cues except stiffness. This approach allowed us to independently investigate the role of stiffness and other HA-derived signals in modulating PDPC differentiation towards the osteogenic phenotype.

The literature is full of studies on the mechanical properties of gels as biomaterials. There are however large discrepancies in the data reported, mainly due to sample state during characterisation (e.g. dry or swollen, pre-stressed or un-stressed)^11^,^13^,^39^. Since the physiological condition involves highly hydrated materials, in this work only fully swollen samples were tested. This approach enables highly reproducible measurements on materials as they are in an equilibrium state^40^. The compressive moduli of HA/Gels (Fig. 1a) were found to be significantly lower than those reported for similar structures tested in the dried state^11^,^13^. However, they were consistent with those recently reported by Kane *et al.*, who tested hydrated HA-collagen scaffolds^21^.

Bone cells are known to be highly sensitive to the physicochemical properties of the substrate, which also have an impact on osteogenic processes. Cell adhesion represents the initial phase of cell-scaffold communication, triggering numerous cellular responses, including proliferation and differentiation. Cells explore the mechanical properties of their immediate microenvironment by generating filopodia, i.e. short cell protrusions that act as biomechanical sensors^41^ and play important regulatory roles in other cellular processes such as cell migration, matrix remodelling and degradation. Here we show that PDPCs cultured on the different substrates differ in cell behaviour and this difference is mainly related to the change of stiffness, confirming their role as mechano-sensing cells. Osteogenesis is in fact dependent on an ordered sequence of gene activation starting with the activation of the bmp2 pathway which usually triggers mRNA runx2 transcription, one of the first factors directing the differentiation of precursor stromal stem cells (i.e. PDPCs) towards an osteoblastic lineage^42^. In cells cultured on HA/Gel and Gel scaffolds we observed an almost comparable sequence of gene activation, confirming the hypothesis that substrate stiffness provides instructive signals guiding cell behaviour. Comparing the gene expression data of PDPCs after 7 days of culture on HA/Gel and Gel scaffolds, we observed that the presence of HA promotes an acceleration in cell differentiation. The expression of all genes involved in osteogenesis was up-regulated on HA/Gel scaffolds, with significant changes starting from the 25/75 HA/Gel scaffold. Remarkably, only in PDPCs seeded on 70/30 HA/Gel scaffolds the increase in bglap expression was associated with a decrease in bmp2 mRNA transcription. This feature was not evident in cells seeded on Gel scaffolds, in which an increase in bglap expression is promoted. The concomitant interchange of bmp2 and bglap in PDPCs seeded on 70/30 HA/Gel scaffolds suggested that these cells were at a further stage of differentiation than on all the other scaffolds. Finally, even though bmp2 and runx2 have been shown to cooperatively interact to stimulate osteoblast gene expression^42^, in our cells bmp2 overexpression is not always associated with an increase of runx2 mRNA levels. This observation strengthens the hypothesis that bmp2 signalling for osteoblast differentiation can also function independently of runx2^43^. Since the osteochondral interface gradually ranges from cartilage to subchondral bone, a functionally graded biomimetic scaffold with various HA/Gel weight ratios reproducing those of cartilage and bone is an attractive option for regeneration of osteochondral tissue^11^,^13^,^35^. To date, most interfacial tissue engineering approaches have used stratified designs, in which there are two or more discrete layers comprising the osteochondral interface^11^,^16^,^44^,^45^. However, discontinuities at the interface between layers may affect pore interconnectivity (with negative effects on cell ingrowth and transport of nutrients and wastes) and can cause layer delamination due to stress concentration. Owing to these limitations, the design of continuously graded structures, with a gradual transition from one tissue type to another, has gained increasing attention - especially in the development of scaffolds for osteochondral tissue regeneration^46^,^47^. We previously designed and validated a method for the fabrication of continuously graded scaffolds based on the sedimentation of HA particles in a HA-gelatin suspension using a controlled gelation rate to obtain different HA particle gradient profiles^12^. The combination of the results obtained in this study with the HA/Gel continuous graded scaffolds could represent a first step towards the generation of the osteochondral interface *in vitro* using PDPCs.

The worldwide demographic shift poses new clinical challenges and stresses the need for innovative approaches to repair tissue in bone aging and/or bone metabolic diseases. Indeed a conclusive correlation between structure and chemistry of the substrate and cell activity is far from being established, particularly in mature microenvironments. Furthermore, despite the growing interest in transplantation of stem cells, their decrease in viability and impaired function with age could hamper clinical outcomes^48^,^49^. In this work we describe an experimental approach for decoupling the contribution of substrate stiffness and other material-related cues in the modulation of stem cell fate. In particular, using periosteal derived progenitor cells seeded on scaffolds with different HA content and their HA-free mechanical equivalents, we show that both stiffness and hydroxyapatite content contribute to modulate osteoblastic-related gene expression in PDPCs. It is worth nothing that the instructive signals promoting the mRNA up-regulation of bmp2, runx2 and bglap in PDPCs came only from the substrate since cells were cultured using a non-differentiating medium (DMEM/F12). Cells cultured on TCP using the same medium did not demonstrate any up-regulation of the investigated osteogenic genes after 7 days (Fig. 3). This suggests that both mechanical and other HA-related signals could be judiciously combined to engineer smart scaffolds supporting osteo-regeneration. Although they did not mimic native tissue stiffness, which has a much higher elastic modulus, the scaffolds with different HA content clearly promoted the up-regulation of the selected gene panel for osteoblastic differentiation of PDPCs. However, our results show that these changes can occur even in the absence of the mineral phase, albeit at a slower rate, suggesting that instructive signals for PDPC bmp2, runx2 and bglap up-regulation derive principally from the substrate stiffness: the higher the substrate stiffness, the higher the modulation of osteogenic stem cell differentiation. The other HA-related cues were found to play a key role in accelerating the gene expression dynamics (i.e. the differentiation rate). Given the role of cell-substrate interactions in directing cell fate, this study represents a key step towards the design of innovative strategies for engineering materials for the replacement of diseased and/or aged tissues. More importantly, we have established a method for decoupling the bulk mechanical properties from other material-derived signals and shown how critical it is to isolate specific cues provided by composite materials for the selective investigation of cell-ECM interactions. Using a strategy similar to that described here, the decoupling concept could be extended to other material properties (e.g. surface chemistry, topography, roughness) to complete the “cause-effect” analysis between substrate features and cell response.

## Methods

### Scaffold fabrication

A 5% w/v solution of gelatin (type-A from porcine skin, G2500, Sigma-Aldrich, Milan, Italy) was dissolved by stirring in 1 × phosphate buffered saline (PBS, Sigma-Aldrich) for 2 h at 50 °C. Hydroxyapatite powder (21223, Sigma-Aldrich) was sieved to obtain particles with diameter < 20 μm. Hydroxyapatite/gelatin (HA/Gel) composites with different composition (i.e. HA/Gel = 0/100, 10/90, 25/75, 60/40, 70/30 w/w) were prepared by adding HA particles to the gelatin solution. These weight ratios mimic those typical of osteochondral tissue, gradually ranging from cartilage (0/100) to subchondral bone (70/30)^11^,^13^,^35^. Each mixture was kept at 50 °C and stirred until a homogeneous HA/Gel suspension was obtained. Then 460 μL of each mixture were poured into individual cylindrical molds (14 mm diameter, 3 mm height) which were immediately transferred in a −20 °C freezer to rapidly form the composite hydrogel scaffolds and prevent the sedimentation of HA particles. HA/Gel scaffolds with a homogeneous distribution of HA were removed from the mold and immersed for 48 h at 4 °C in a crosslinking solution of 5 mM GTA in 40% ethanol (EtOH, Sigma Aldrich), keeping a constant 5:1 volume ratio between the GTA solution and the HA/Gel samples.

HA-free Gel scaffolds were crosslinked using solutions with different concentrations of GTA. Firstly, different samples were prepared in order to find the mathematical relation between GTA concentration and scaffold stiffness. Four different GTA concentrations (i.e. 5, 10, 50, 100 mM, all well below the GTA levels associated with toxicity^32^) were investigated. Then, using the mathematical relationship between GTA concentration and stiffness, appropriate crosslinking solutions were chosen and used to prepare samples with stiffness matching those of HA/Gel samples (see Results section for further details).

All samples were finally washed thrice with PBS 1×, soaked in a 0.1 M glycine solution for 2 h at room temperature to quench any residual aldehyde moieties^50^, rinsed twice with PBS and stored in a 40% v/v EtOH solution.

### Mechanical testing

Samples were tested in unconfined uniaxial compression at a fixed strain rate of 0.01 s^−1^ using a twin column Zwick/Roell ProLine Z005 testing device (Zwick/Roell, Ulm, Germany) equipped with a 10 N load cell. Mechanical tests were performed at room temperature, maintaining samples partially immersed in PBS 1× to preserve their hydration during experiments^21^,^39^,^40^. Prior to testing, each sample was carefully measured in thickness (*l*_*0*_) and diameter (*d*). Measurements were performed with a calliper (0.05 mm resolution), averaging readings from at least three different points. Force (*F*) and displacement data (*l*) were recorded starting with a zero stress initial condition, as described in Tirella *et al.* and Mattei *et al.*^39^,^40^. Force and actual compressive displacement data were respectively normalised to the cross-sectional area (*πd*^2^/4) and the initial length of the sample (*l*_*0*_), obtaining the engineering stress (σ) and strain (ε). Compressive moduli were derived as the slope of the first linear portion of the stress-strain plot^13^,^16^.

### Human periosteal derived precursor cells (PDPCs) harvesting

Periosteal tissue was obtained from 6 subjects (4 male and 2 female, mean age 34 years) undergoing surgery for orthopaedic trauma, as previously described^28^,^51^. All patients provided their informed consent to participate in the study. Since the study did not expose the subjects to any risk, in lieu of a written consent form, a verbal authorization was obtained from all the recruited patients, in accordance with the Ethical Committee guidelines of the Università Politecnica delle Marche. It was underlined to all subjects that the tissue used for the study represents the typical discard during the surgical procedures and that the nature of their participation in the study was entirely voluntary (freedom from coercion or undue influence, real or imagined). Patients had sufficient opportunity to ask questions and consider their choice. Approval from the local Ethical Committee of the Università Politecnica delle Marche is not required for the use of waste tissue after informed consent has been obtained. Briefly, periosteal tissue was aseptically dissected, washed three times in Dulbecco’s Phosphate-Buffered Saline (D-PBS) lacking in Ca^2+^ and Mg^2+^, cut into small pieces (2-3 × 2-3 mm) and placed in a 100 mm culture dish in Dulbecco’s Modified Eagle Medium/Nutrient Mixture F-12 (DMEM/F-12) supplemented with 10% Foetal Bovine Serum (FBS) and 1% penicillin-streptomycin (100 U/mL)^52^. Both culture media and supplements were purchased from GIBCO^®^ (Life Technologies, Milan, Italy). The medium was changed twice weekly and cells were used between the 3^rd^ and the 6^th^ passage of subculture.

Cell characterisation According to The International Society for Cellular Therapy for identification of human MSCs^53^, PDPCs were analysed by a FACS Calibur flow cytometry system (Becton Dickinson, CA, USA) and subjected to differentiation into osteoblastic lineage. For immunophenotyping 2.5 × 10^5^ cells were removed with D-PBS and then stained for 45 min with the following antibodies: fluorescein isothiocyanate-(FITC)-labeled mouse anti-human CD90 (StemCell Technologies, Milan, Italy), CD105, CD14, CD19 (Diaclone, France), and R-phycoerythrin-(PE)-labeled mouse anti human CD34, CD45 (Diaclone, France), CD73 (Becton Dickinson) and anti HLA-DR purchased from Diaclone. The control for FITC or PE coupled antibodies was an isotypic mouse IgG1, while for un-conjugated antibodies secondary FITC-labelled goat anti-mouse IgG was used as a control. Data were evaluated using the CellQuest software (Becton Dickinson).

Cell differentiation was performed using STEMPRO® Osteogenesis Kits (Life Technologies Corporation, USA) according to the manufacturer’s instructions. In brief cells were seeded at a density 4.5 × 10^4^cells with appropriate medium for 7 days, changing the medium twice a week. Osteogenic differentiation was assessed by Von Kossa and Alkaline phosphatise (ALP) staining. For Von Kossa staining, cells were fixed in 4% Paraformaldehyde (PFA) for 15 min at room temperature (RT) and incubated with 1% silver nitrate solution under UV light for 20 min RT. Unreacted silver was removed with 5% sodium thiosulfate for 5 min. For ALP staining, cells were fixed in 4% PFA for 15 min RT and washed in 100 mM Tris-HCl pH 9.5, 100 mM NaCl and 10 mM MgCl_2_ buffer for 10 min RT. Cells were then stained with fast 5-bromo-4-chloro-3-indolyl phosphate and nitroblue tetrazolium alkaline phosphate substrate (Sigma-Aldrich, Milan, Italy) for 10 min and rinsed in dH_2_O. The reaction was observed with a light microscope (Nikon Eclipse 600, Nikon, Milan, Italy). Cells cultured in DMEM/F-12 with 10% FBS were used as negative controls.

### Cell seeding

Before seeding, all the scaffolds (HA/Gel and Gel scaffolds) were sterilized in 70% v/v EtOH for 2 h, washed two times in PBS 1 × (GIBCO) for 30 min and placed under UV light for 30 min each side. They were then placed in Corning^®^ ultra-low attachment multiwell plates and conditioned overnight in DMEM/F-12 with 10% serum (i.e. non-differentiating culture medium) in an incubator (5% CO_2_, 37 °C). The medium was then discarded and scaffolds ready for seeding. Undifferentiated PDPCs were detached using 0.25% trypsin in 1 mM ethylenediaminetetraacetic acid (EDTA, Sigma-Aldrich) and seeded at a density of 1 × 10^4^  cell/cm^2^ by applying 50 μL of cell suspension on the samples. After 30 min in an incubator the samples were covered by 1.5 mL of DMEM/F-12 and cultured for 2 and 7 days. The intermediate incubation step was used to ensure the cells adhered to the scaffold without sliding off and falling in the wells.

### Scanning electron microscopy (SEM) and micro-analytical investigation (EDX)

Scaffolds were observed with a Philips XL 20 SEM (FEI Italia SRL, Milan, Italy) equipped for X-ray microanalysis (EDS-PV 9800). Specimens were observed using the secondary electron (SE) detector. The EDX control program was used for collecting spectra and EDX mapping was applied to determine elemental distribution. Operating conditions for the analysis were: voltage 20 KV, magnification 500×, counts per second (cps) no less than 2.000, tilt angle 15°, time count 250 s. Only the Kα values of each element were considered, and the semi quantitative percentage concentrations were calculated using the ZAF (Z = atomic number; A = adsorption; F = fluorescence) procedure correction.

For SEM analysis after 2 and 7 days of cell culture, the samples were fixed in 2% v/v GTA in 0.1 M cacodylate buffer (pH 7.4), post-fixed in 1% v/v osmium tetroxide, dehydrated in increasing ethanol concentrations, critical point dried, mounted on aluminium stubs and gold-sputtered.

### RNA extraction, quantitation and reverse transcription

After 7 days of culture total RNA was isolated from PDPCs with TRIzol^®^ Reagent (Invitrogen, Milan, Italy), according to the manufacturer’s instructions. Quantification and evaluation of RNA quality were performed by spectrophotometric analysis (bioPhotometer plus, Eppendorf GmbH, Germany). One microgram of total RNA was reverse transcribed in a 20 μL reaction volume using the GoScript™ Reverse Transcription System (Promega, Milan, Italy). Neo-synthesized cDNA was stored at −20 °C.

### RT-qPCR assay

Real-time assays were performed using a Mastercycler Realplex2 thermocycler (Eppendorf GmbH, Germany) with SsoFast™ EvaGreen^®^ Supermix 1×, in a final volume of 10 μL. All PCR reactions contained 1 μL of cDNA (corresponding to 50 ng of total RNA template). Each PCR assay was performed in white plastic-ware and comprised 30 seconds at 95 °C for enzyme activation, 40 cycles of denaturation at 95 °C for 5 seconds, annealing and extension at 60 °C for 20 seconds. Each primer was used at a 200 nM final concentration. Primer sequences were designed by Primer 3 (v. 0.4.0) software and their specificity was tested by BLAST Assembled RefSeq Genomes in order to avoid any appreciable homology to pseudo-genes or other unexpected targets. The oligonucleotide sequences for target genes are listed in Table 1.

In each assay, both reference genes and each gene of interest mRNA were measured simultaneously under identical conditions. Primers showed the same amplification efficiency. Specificity of the PCR reactions was furthermore confirmed by melt curve analysis.

### Quantification of mRNA expression

Each assay was performed as triplicate and reference gene’s Cq values were used to normalise cellular mRNA data. In this instance, normalisation involved the ratio of mRNA concentrations of specific genes of interest (as mentioned above) to that of the GAPDH Cq medium value. In order to highlight the effect of mechanical stimuli on PDPCs, the ΔΔCq method for the evaluation of Fold-Change was employed using cells seeded on 0/100 structures as an internal control. The relative amount of each mRNA was calculated using the comparative threshold (Ct) method with ΔCt = Ct(mRNA) − Ct(GAPDH) and relative quantification of mRNA expression was calculated with the 2^−ΔΔCt^ method^54^. The qPCR efficiency in all our experiments was more than 90%. The difference between the actual and theoretical (100%) efficiencies would result in an underestimation of the mRNA concentration of all analysed samples.

Data in histograms are expressed as fold-regulation that represents fold-change results in a biologically meaningful way. In particular, fold-change values greater than one indicate an up-regulation, and the fold-regulation is equal to the fold-change. Fold-change values less than one indicates a down-regulation, and the fold-regulation is the negative inverse of the fold-change.

### Statistical analysis

Experiments were all performed in triplicate. Data are reported as mean ± standard deviation, unless otherwise noted. Data were analysed by one-way ANOVA, Student-Newman-Keuls’s and Student’s t-tests. Statistical significance was set at *p* < 0.05.

## Additional Information

**How to cite this article**: Mattei, G. *et al.* Decoupling the role of stiffness from other hydroxyapatite signalling cues in periosteal derived stem cell differentiation. *Sci. Rep.* **5**, 10778; doi: 10.1038/srep10778 (2015).

## Supplementary Material

Supplementary Information

## Figures and Tables

**Table 1:** Analysed gene description.

**Genes**	**Detected Transcript**	**Primer Forward (5’−>3’)**	**Primer Reverse (5’−>3’)**	**Amplicon length (bp)**	**Annealing T (°C)**
**BMP2**	**NM_001200.2**	CCAGCCGAGCCAACACTGTGC	TCTCCGGGTTGTTTTCCCACTCG	86	60°
**RUNX2**	**NM_004348.3**	CTCGTCCGCACCGACAGCC	TACCTCTCCGAGGGCTACCACC	111	60°
**BGLAP**	**NM_199173**	GACTGTGACGAGTTGGCTGA	GCCCACAGATTCCTCTTCTG	119	64°
GAPDH*	**NM_002046.3**	AGCCACATCGCTCAGACAC	GCCCAATACGACCAAATCC	200	60°

^*^reference gene
