# Corrigendum: Decoupling the role of stiffness from other hydroxyapatite signalling cues in periosteal derived stem cell differentiation

**DOI:** 10.1038/srep14590

**Published:** 2015-10-20

**Authors:** Giorgio Mattei, Concetta Ferretti, Annalisa Tirella, Arti Ahluwalia, Monica Mattioli-Belmonte

Scientific Reports 5: Article number: 10778; 10.1038/srep10778 published online: 06022015; updated: 10202015.

In this Article, the legend of Fig. 6 contains typographical errors.

“Changes in bmp2, runx2 and osteocalcin (bglap) mRNA expression in PDPCs seeded on (**a**) Gel and (**b**) HA/Gel scaffolds. Data are expressed as fold-regulation which represents fold-change results in a biologically expressive manner. Fold-regulation is equal to the fold-change (2^−ΔΔCt^) for fold-change values greater than one, which indicate an up-regulation. Fold-change values less than one indicate a down-regulation: in this case the fold-regulation is the negative inverse of the fold-change (−1/2^−ΔΔCt^). a = *p* < 0.05 vs CTRL; b = *p* < 0.05 vs 25/75 scaffolds; c = *p* < 0.05 vs 60/40 scaffolds.”

should read:

“Changes in bmp2, runx2 and osteocalcin (bglap) mRNA expression in PDPCs seeded on (**a**) HA/Gel and (**b**) Gel scaffolds. Data are expressed as fold-regulation which represents fold-change results in a biologically expressive manner. Fold-regulation is equal to the fold-change (2^−ΔΔCt^) for fold-change values greater than one, which indicate an up-regulation. Fold-change values less than one indicate a down-regulation: in this case the fold-regulation is the negative inverse of the fold-change (−1/2^−ΔΔCt^). * = *p* < 0.05 vs CTRL; # = *p* < 0.05 vs 25/75 scaffolds; § = *p* < 0.05 vs 60/40 scaffolds.”

