# Supplementary material for: Decoupling the role of stiffness from other hydroxyapatite signalling cues in periosteal derived stem cell differentiation
Source: Sci Rep. 2015 Jun 2;5:10778. doi: 10.1038/srep10778 (PMC4451686; doi:10.1038/srep10778)
Supplement: Supplementary Information [file srep10778-s1.pdf]

# Decoupling the role of stiffness from other hydroxyapatite signalling cues in periosteal derived stem cell differentiation

Giorgio Mattei, Concetta Ferretti, Annalisa Tirella, Arti Ahluwalia, Monica Mattioli-Belmonte

## SUPPLEMENTARY INFORMATION

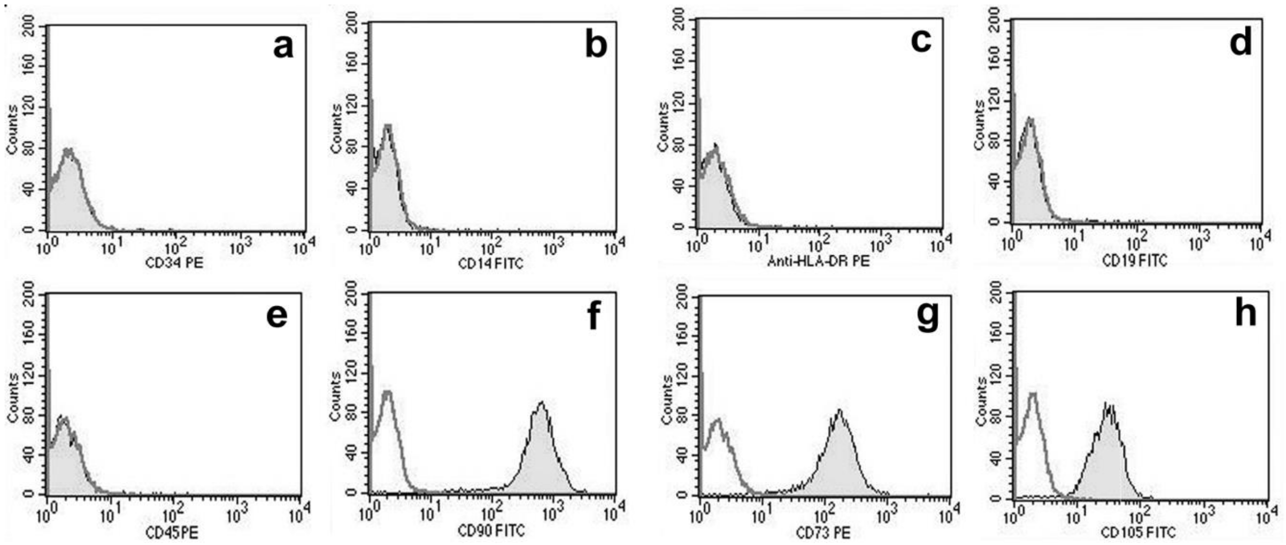


**Supplementary Fig. 1.** PDPCs phenotypic profile. Cells showed a negative reaction for CD34, CD14, HLA-DR, CD19 and CD45 (**a-e**) and cell positivity for CD90 (**f**), CD73 (**g**) and CD105 (**h**), which are the typical mesenchymal stem cell (MSC) markers.

### SI 1. AFM topographical imaging

A commercial AFM (Agilent 5500 ILM AFM, Agilent Technologies, Milan, IT) mounted on an inverted optical microscope (Olympus IX70, Olympus, Milan, IT) was used to determine the surface roughness of Gel substrates. All samples were imaged in contact mode in liquid using DNP-S10 cantilevers (VEECO Instruments, Woodbury, NY) with a nominal spring constant of 0.06 N/m,

acquiring scans of 512×512 lines over an area of 5 µm × 5 µm[*]. A representative image of a Gel sample is shown in Supplementary Fig. 2.

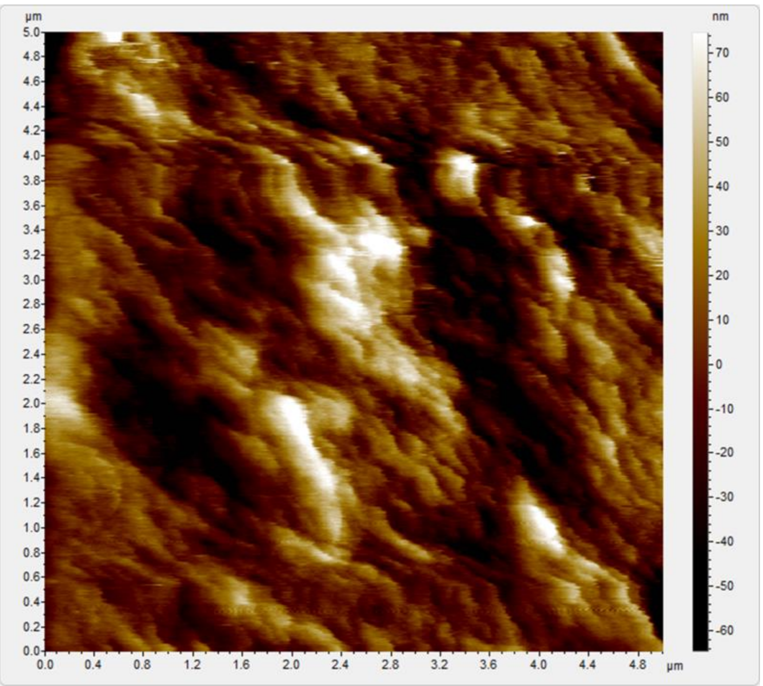


**Supplementary Fig. 2.** Topographic surface profile of 34 mM Gel substrate obtained using Atomic Force Microscopy (AFM) in liquid.

The root mean square (RMS) roughness ($R_q$) was evaluated according to the following calculation implemented in Matlab® (The Mathworks Inc., Natick, MA, USA):

$$R_q = \sqrt{\frac{1}{n}\sum_{i=1}^{n} y_i^2} \qquad (1)$$

where $y_i$ denotes the vertical distance from the mean line to the $i^{th}$ data point, and $n$ represents the number of equally spaced points experimentally measured along the line[1].

[*] AFM measurements were performed at the Department of Biophysical and Electronic Engineering of the University of Genova (Italy) by Prof. Roberto Raiteri and Dr. Leonardo Peñuela of the Neuroengineering and Bio-NanoTechnology Group to whom we are grateful.

A total of 3 locations were analysed for each of the 5 Gel samples, and the average RMS roughness compared using one-way ANOVA followed by Tukey's post hoc test setting statistical significance at $p < 0.05$.

No significant differences in $R_q$ were found across Gel samples, with an average $R_q = 31.1 \pm 13.3$ nm. These results are consistent, both in terms of values and trend, with those published by Flores-Merino et al. who tested 1-vinyl-2-pyrrolidone (PVP) hydrogels at different concentrations of di-ethylene glycol bis-allyl carbonate (DEGBAC) crosslinker exhibiting very similar mechanical properties to those of our gels, reporting no statistically differences in their surface roughness[2]. Eroshenko et al. recently reported that surface roughness does not change significantly across PDMS substrates (Sylgard 184, Dow Corning, Midland, MI) prepared at different base to catalyst ratios[3], further suggesting that this parameter is insensitive to crosslinker concentration.

The roughness of HA/Gel substrates is expected to be different from that of Gel substrates due to the presence of HA particulate and likely to increase with the inorganic phase concentration[4]. Unfortunately, no AFM topographical images of HA/Gel substrates were obtained in liquid due to experimental limitations related to the presence of the inorganic filler (dynamic mode was also investigated, but with no results). It is worth noting that the vast majority of papers report AFM imaging of HA-containing samples under dry conditions (i.e. in air)[5–8], probably due to the difficulty of performing imaging "in liquid". Since this state is poorly representative of that experienced by cell during culture, AFM imaging in air was not considered in this work. However, this is not a major limitation to the study since surface roughness of HA/Gel substrates is part of the other HA-related signals that were not individually studied in this work.

## References


1. Gadelmawla, E. S., Koura, M. M., Maksoud, T. M. A., Elewa, I. M. & Soliman, H. H. Roughness parameters. *J. Mater. Process. Technol.* **123,** 133–145 (2002).

2. Flores-Merino, M. V. *et al.* Nanoscopic mechanical anisotropy in hydrogel surfaces. *Soft Matter* **6,** 4466 (2010).

3. Eroshenko, N., Ramachandran, R., Yadavalli, V. K. & Rao, R. R. Effect of substrate stiffness on early human embryonic stem cell differentiation. *J. Biol. Eng.* **7,** 7 (2013).

4. Kim, H.-W., Kim, H.-E. & Salih, V. Stimulation of osteoblast responses to biomimetic nanocomposites of gelatin-hydroxyapatite for tissue engineering scaffolds. *Biomaterials* **26,** 5221–30 (2005).

5. Lekakou, C., Lamprou, D., Vidyarthi, U., Karopoulou, E. & Zhdan, P. Structural hierarchy of biomimetic materials for tissue engineered vascular and orthopedic grafts. *J. Biomed. Mater. Res. B. Appl. Biomater.* **85,** 461–8 (2008).

6. Jaiswal, A. K., Chhabra, H., Soni, V. P. & Bellare, J. R. Enhanced mechanical strength and biocompatibility of electrospun polycaprolactone-gelatin scaffold with surface deposited nano-hydroxyapatite. *Mater. Sci. Eng. C. Mater. Biol. Appl.* **33,** 2376–85 (2013).

7. Li, J. *et al.* Surface characterization and biocompatibility of micro- and nano-hydroxyapatite/chitosan-gelatin network films. *Mater. Sci. Eng. C* **29,** 1207–1215 (2009).

8. Katti, K. S., Katti, D. R. & Dash, R. Synthesis and characterization of a novel chitosan/montmorillonite/hydroxyapatite nanocomposite for bone tissue engineering. *Biomed. Mater.* **3,** 034122 (2008).
